# Analysis of Trafficking, Stability and Function of Human Connexin 26 Gap Junction Channels with Deafness-Causing Mutations in the Fourth Transmembrane Helix

**DOI:** 10.1371/journal.pone.0070916

**Published:** 2013-08-15

**Authors:** Cinzia Ambrosi, Amy E. Walker, Adam D. DePriest, Angela C. Cone, Connie Lu, John Badger, I. Martha Skerrett, Gina E. Sosinsky

**Affiliations:** 1 National Center for Microscopy and Imaging Research, Center for Research in Biological Systems, University of California San Diego, La Jolla, California, United States of America; 2 Department of Neurosciences, University of California San Diego, La Jolla, California, United States of America; 3 Biology Department, State University of New York Buffalo State, Buffalo, New York, United States of America; 4 DeltaG Technologies, San Diego, California, United States of America; Emory University School of Medicine, United States of America

## Abstract

Human Connexin26 gene mutations cause hearing loss. These hereditary mutations are the leading cause of childhood deafness worldwide. Mutations in gap junction proteins (connexins) can impair intercellular communication by eliminating protein synthesis, mis-trafficking, or inducing channels that fail to dock or have aberrant function. We previously identified a new class of mutants that form non-functional gap junction channels and hemichannels (connexons) by disrupting packing and inter-helix interactions. Here we analyzed fourteen point mutations in the fourth transmembrane helix of connexin26 (Cx26) that cause non-syndromic hearing loss. Eight mutations caused mis-trafficking (K188R, F191L, V198M, S199F, G200R, I203K, L205P, T208P). Of the remaining six that formed gap junctions in mammalian cells, M195T and A197S formed stable hemichannels after isolation with a baculovirus/Sf9 protein purification system, while C202F, I203T, L205V and N206S formed hemichannels with varying degrees of instability. The function of all six gap junction-forming mutants was further assessed through measurement of dye coupling in mammalian cells and junctional conductance in paired Xenopus oocytes. Dye coupling between cell pairs was reduced by varying degrees for all six mutants. In homotypic oocyte pairings, only A197S induced measurable conductance. In heterotypic pairings with wild-type Cx26, five of the six mutants formed functional gap junction channels, albeit with reduced efficiency. None of the mutants displayed significant alterations in sensitivity to transjunctional voltage or induced conductive hemichannels in single oocytes. Intra-hemichannel interactions between mutant and wild-type proteins were assessed in rescue experiments using baculovirus expression in Sf9 insect cells. Of the four unstable mutations (C202F, I203T, L205V, N206S) only C202F and N206S formed stable hemichannels when co-expressed with wild-type Cx26. Stable M195T hemichannels displayed an increased tendency to aggregate. Thus, mutations in TM4 cause a range of phenotypes of dysfunctional gap junction channels that are discussed within the context of the X-ray crystallographic structure.

## Introduction

Connexins are a family of membrane proteins expressed in most tissues of higher vertebrates. Twenty-one different human connexin genes have been reported so far, each coding for a transmembrane protein with the same protein topology [1]. Connexins are known for their ability to form hexamers in the plasma membrane. These hexameric assemblies (hemichannels or connexons) dock at their extracellular membrane and assemble together, forming intercellular channels. Note that here we use connexon and hemichannel synonymously to indicate the connexin hexamer (c.f. editorial preface in Harris and Locke [2]). Clusters of intercellular channels in the apposing area between two cells are called gap junctions [3]. A connexin consists of four α-helical transmembrane domains (TM1–TM4), two extracellular loops (EL1 and EL2), a cytoplasmic loop (CL) between TM2 and TM3, and cytoplasmic amino -terminal (NT) and carboxy -terminal (CT) domains [4]. Current crystallographic structures have revealed that the N-terminus is part of the pore entrance structure [5]–[8]. Connexin proteins are named after their predicted molecular weight from the gene sequence in kDa (for instance, Cx26 has a calculated molecular mass of ∼26 kDa). Their genes have been classified into 4–5 groups (α, β, γ, δ and sometimes ε) based on sequence homology and named accordingly (for example Cx43, which is the first connexin of the α-group, is coded by GJA1, http://www. genenames.org/gene family/gj.php).

Connexins play a broad spectrum of roles in several tissues. Consequently, mutations in their sequences have been associated with numerous diseases [9]. The most widely studied example of connexin-associated disease has been Cx26 (GJB2) in hearing loss [10]. Mutations in human Connexin26 (**hCx26**) can lead to congenital hearing loss (1 child per 1000 frequency) [11] that can be syndromic or non-syndromic. Non-syndromic hearing loss (**NSHL**) is characterized by sensorineural hearing loss in the absence of other symptoms, while syndromic hearing loss affects other organ systems, primarily the skin. The gene sequences linked to non-syndromic hearing loss could be categorized into dominant (DFNA), recessive (DFNB), X-linked (NFDX) and Y-linked (NFDY). Over 80 different genes have been linked to non-syndromic hearing loss, covering a large spectrum of molecules critical for the normal function of the ear, however mutations in GJB2 (the gene that encodes for Cx26) account for about half of all congenital and autosomal recessive nonsyndromic hearing loss in every population tested [10], [12]. Although the most frequently occurring NSHL mutations produce severely truncated proteins due to frameshift or missense, almost 80% of the known deafness mutations are actually single amino acid changes or deletions. These mutations have been found across the entire sequence of Cx26.

The majority of NSHL mutations cause either generalized folding problems that result in the failure of Cx26 to traffic to the cell surface, or are permissive for the formation of gap junction plaques, but prevent intercellular channel function. As Xu and Nicholson [10] pointed out in a recent review, there are no specific “hot spots” for NSHL single amino acid mutations within the Cx26 sequence. In general, single site mutations are spread fairly evenly across the whole protein with TM2 having the highest mutation density (number of amino acids with NHLS mutations divided by the total number of amino acids in the domain) at 67% to M1 and E1 having the lowest density of mutations with their respective domains at 33%. According to this criterion, TM4 has a mutation density of 40%. Nonetheless, it should be noted that domain boundaries in the Xu and Nicholson analysis [10] are approximate because there is no membrane in the X-ray crystallographic structure [5] and the membrane bilayer is not well-resolved in electron microscopy (**EM**) crystallographic structures [7], [8], [13].

Of the four transmembrane helices, M1, M2 and M3 have attracted the most attention, because of the controversies involved in models with different helix assignments, based on lower resolution cryo-electron crystallographic structures and scanning cysteine accessibility mutagenesis (SCAM, see [14] for further discussion). Far less is known about TM4 and how side chains interact with the other helices and with the lipid bilayer. Our published 5Å EM crystallographic structure of the mutant Cx26M34A within a lipid membrane revealed an outward expansion of all four transmembrane helices and a closure of the channel relative to the X-ray crystallographic structure of wild-type (**WT**) Cx26 channels in detergent [8]. For this reason, we have been particularly interested in determining changes of the structure in TM4 helix mutations and in defining mechanisms, by which the movements of these helices are coordinated during opening and closing of the channel. While point mutations in the TM4 region affect the correct folding of Cx26 and its functionality [10], [15], more recent studies on Cx26 emphasized the role of some specific “key residues” [16]–[20].

Here we confirmed these observations, analyzing the stability of hemichannels with point mutations in the TM4 region that are linked to NSHL. Specifically, we characterized fourteen point deafness mutations at twelve amino acids along the entire stretch of TM4 for proper trafficking and function. Two amino acid positions had two different substitutions. Using the Sf9 insect cells-baculovirus expression system, we expressed and purified each of the six Cx26 TM4 mutated proteins that formed gap junction hemichannels and channels and analyzed their oligomeric stability. In the case of unstable mutants, we determined whether co-expression with the WT rescued their hexameric structure as we have shown for other mutants in TM1-3 [21]. Importantly, we also correlated this analysis with tests for functionality of these mutants, both by dye transfer assays and electrophysiological measurements in the paired oocyte system.

## Materials and Methods

### Generation of connexin constructs and site-directed mutagenesis

Unless identified otherwise, all mutants and WT species tested in this study are human Cx26. We generated Cx26 point mutations using the QuikChange site-directed mutagenesis kit (Stratagene, La Jolla, CA) on a previously cloned WT construct in pcDNA3.1 [7], [22]. For mammalian constructs, Cx26 mutant DNAs were then inserted into a pIRESneo2 vector that provides a more efficient transfection than pcDNA3.1 and these same constructs were used for analyzing the mutants' ability to form gap junctions and to pass the dye. For baculovirus expression, Cx26 constructs were all inserted into the pBlueBac4.5 vector or in the pCR8/GW/Topo2.8 vector (Invitrogen, Carlsbad, CA). These plasmids incorporate a hexahistidine (**His_6_**) tag with a thrombin recognition sequence (LVPRGS) fused to the C terminus of human Cx26 to enable protein purification by nickel-affinity chromatography, as well as a V5 epitope (GKPIPNPLLGLDST) for protein identification. In addition, WT was also cloned into the baculovirus construct with the V_5_- His_6_ tag for membrane purification experiments. It should be noted that the V_5_- His_6_ tag does not affect the Cx26 structure as demonstrated by the fact that the X-ray crystals that were produced with a protein that had no hexahistidine tag [5]. It is essentially the same structure as the EM crystal structure from our laboratory that retained the hexahistidine tag [7], [8].

### Baculovirus Expression of Cx26 WT and mutants in insect cells

Spodoptera frugiperda (Sf9) cells were cultured in the Sf900III-SFM media supplemented with 2% fetal bovine serum and 0.1% antibiotic/antimycotic (Invitrogen) at 27°C. Cells were infected at a density of 1.8×10^6^ cells/ml for expression and then recovered 72 h after infection. The Multiplicity Of Infection (**MOI**) of each baculovirus was calculated applying a protocol where Sf9 cells were infected according to the Bac-N-Blue Transfection and Expression Guide Invitrogen manual with each mutant baculovirus. The infection was performed for 72 hours at 28 degrees. Infected cells were then fixed in 4% Paraformaldehyde (PFA), washed in Dulbecco's Phosphate Buffered Saline 1X (DPBS 1X) buffer and stained with 0.1% Neutral Red. The staining was performed for at least 30 minutes. After several washes with DPBS, the cells were analyzed by inverted microscope. At least five fields of view were considered, where the total number of cells was counted as well as the dead cells (cells that were infected appearing white). The percentage of dead cells on the total cells was calculated and then averaged for all the five fields of view considered. Dead cells coming from non-infected plates were considered as background and subtracted from the measured infected plates.

### Hemichannel purification and identification

Hemichannels were isolated from baculovirus-infected Sf9 cells according to our published methods [21]. Briefly, solubilized hemichannels were prepared from isolated Sf9 membranes by incubation in 2% dodecyl maltoside in HEPES buffer (10 mM HEPES, 200 mM NaCl, pH 7.4) and affinity purified using the C-terminal His_6_ tag and nickel-nitrilotriacetic acid–agarose (Ni-NTA). For rescue experiments, baculovirus-expressed mutant Cx26 proteins contained a V5-His_6_ tag for affinity purification while the WT baculovirus did not [21]. Cells were co-infected with mutant hCx26-V5-His6 and WT in varying ratios of MOIs as described in [21].

### Blue Native (BN) gel PAGE and Western blots

Blue Native gel electrophoresis was performed using 4–20% gradient gel (NativePAGE Novex Bis-Tris Gel System, Invitrogen, Carlsbad, CA). For transferring the proteins on PVDF membrane (Millipore, Billerica, MA), we used NuPAGE transfer buffer and the iBlot transfer apparatus (Invitrogen, Carlsbad CA). Bands on Western blots were identified using the mouse monoclonal anti-His (C-term) (Invitrogen, Carlsbad CA) and visualized with enhanced chemiluminescence Western blotting procedures, using SuperSignal West Pico (Thermo Scientific, Waltham, MA) or Luminata Forte (Millipore, Billerica, Ma).

### Light microscopy

Confocal images were acquired using a Leica TCS SPE confocal microscope with a 20X oil immersion objective lens (Leica Microsystems Inc., Buffalo Grove, IL) or an Olympus Fluoview1000 microscope (Olympus USA, PA).

### Electron Microscopy and Image Processing Procedures

Samples were diluted 2–10x with ddH_2_O and negatively stained with 2% uranyl acetate for electron microscopic observation. Electron micrographs were recorded on a JEOL 1200 120 keV (JEOL, Peabody, MA) or an FEI Spirit 120 keV microscope at an accelerating voltage of 80 keV and an instrument magnification of 30 KX.

### Mammalian Cell Culture and Transfections

HeLa cells were maintained at 37°C and 10% CO_2_ in Dulbecco's modified Eagle's medium (Cellgro, Manassas, VA), containing 10% fetal bovine serum (Gemini, West Sacramento, CA). The HeLa cell line used was a gift from Dr. Bruce Nicholson, University of Texas Health Sciences Center, San Antonio TX and was originally described in Elfgang et al. [23]. Transfections were carried out using Lipofectamine 2000 (Invitrogen) or Amaxa Nucleofector™ 2S device (Lonza, Walkersville, MD) program I-013. For mammalian cell light microscopy, we used Cx26 proteins with the GFP-4C tag genetically appended to a six amino acid linker (PSKLAT) at the C-terminal end of Cx26. The 4C is the tetracysteine domain FLNCCPGCCME [24] and is preceded by the linker ESSGS between the GFP C-terminus and the N-terminus of the 4C domain. The tetracysteine domain is another type of imaging tag we have previously used for correlated light and EM as well as optical pulse chase labeling [25].

### Scrape Loading and Dye Transfer Assay

Procedures were adapted from references [26] and [27] with the following modifications. HeLa cells were transfected at high expression levels, with each of our WT and mutant pIRESneo2-GFP-4C constructs using the Nucleofector™ 2S device. After 48 hours the media was recovered from the cells and kept warm. The cells were washed three times with HBSS buffer with 1% BSA (HBC). A solution of 1X DPBS containing 0.5% of Lucifer Yellow (Invitrogen) and 0.5% of Dextran Texas Red (Invitrogen, Carlsbad CA) was added to cover the cells. Several parallel scratches were performed on the cover slips using a 30 G1/2 needle (Cat # 305106 from BD Inc., Franklin Lakes, NJ) in dye solution, where the cells were left for 1 minute. After this, the cells were washed three times with HBC and put back in the original media for 8 minutes to let the dye pass through the gap junction and diffuse to the neighboring cells. Three more washes in HBSS buffer were performed and then the cells were fixed for 20 minutes in 4% PFA. After 3 more washes in DPBS, the cover slips were mounted on microscope slides with gelvatol for microscopy analysis. Measurements of images along the scrape were performed on 10 images per sample. Only Dextran Texas Red cells along the scratch were counted as non-transferring cells, since the ones far from the scratch were typically cells that died during the exogenous expression process.

### Functional expression in Xenopus oocytes

All work with *Xenopus* frogs was carried out in accordance with recommendations and approval of the IACUC Committee, SUNY Buffalo State. All surgeries were recovery surgeries, performed under Tricaine methanesulfonate (MS-222) anesthesia and all efforts were made to minimize suffering.

The gene for human Cx26 was obtained in the vector pcDNA3.1 with six deafness mutants (M195T, A197S, C202F, I203T, L205V, N206S) encoded. The GFP and tetracysteine tags were removed prior to expression in oocytes via insertion of a stop codon at position 227. Mutagenesis was performed using the Quikchange Lightning protocol (Agilent Technologies, Santa Clara CA) and confirmed by sequence analysis (Roswell Park Cancer Institute, Biopolymer Resource Center, Buffalo, NY). RNA was synthesized using the mMessage mMachine T7 protocol (Life Technologies, Grand Island, NY). RNA was quantified by gel electrophoresis, using an RNA 250 standard (Ambion Life Technologies, Grand Island, NY).

After isolation from adult female *Xenopus* laevis frogs, oocytes were treated with collagenase (Type 1A, Sigma-Aldrich Corp. St. Louis MO) in OR2 media (Oocyte Ringers; 82.5 mM NaCl, 2 mM KCl, 1 mM MgCl_2_, 5 mM HEPES, pH 7.4). Following collagenase treatment, oocytes were rinsed in MB1 (Modified Barth's; 88 mM NaCl, 1 mM KCl, 0.41 mM CaCl_2_, 0.82 mM MgSO_4_, 1 mM MgCl_2_, 0.33 mM Ca(NO_3_)_2_, 20 mM HEPES, pH 7.4) several times and then stored in MB1 at 18°C.

Fine forceps were used to remove remaining follicle cells before a *morpholino* antisense oligonucleotide (GeneTools LLC, Philomath OR) directed against XeCx38 RNA was injected at 0.5 ng per oocyte to prevent expression of endogenous Cx38. The following day, RNA for each mutant and WT Cx26 was injected at a volume of about 40 µl and a concentration of 125 ng/µl, totaling approximately 4 ng/oocyte. After 8–12 hours the vitelline layer was removed with fine forceps and oocytes were paired in agar wells at 18°C overnight. The following day, dual cell two electrode voltage clamp was performed as described by Skerrett et al. [28] to assay the presence and properties of gap junction channels. Data was collected and analyzed using the PClamp 10.2 Suite of software (Molecular Devices, Sunnyvale, CA). Data was further analyzed and plotted using OriginLab (Northampton, MA).

Three protocols were used to assess function. For rapid analysis of junctional conductance, short one second pulses to transjunctional voltages (Vj) of ±100 mV were used. Both cells were initially clamped at −20 mV, junctional current was then measured within the first 100 ms of the Vj change in the continuously clamped cell. For detailed characterization Vj-sensitivity, a series of voltage steps was used. Both cells were initially clamped at −20 mV and voltage steps were applied in 10 mV increments to ±100 mV. In experiments where hemichannels were assayed, the vitelline layer was not removed. Hemichannels were studied in the same batch of oocytes, on the same day as gap junction channels to ensure equal times for expression. Using a voltage-pulse protocol that stepped Vm (transmembrane voltage) from the holding potential (−40 mV) to +60 mV and −160 mV in 20 mV increments. Each voltage pulse lasted two seconds, with 10 second recovery periods between pulses.

### Computational analysis, modeling and graphics

**Figure 1:**
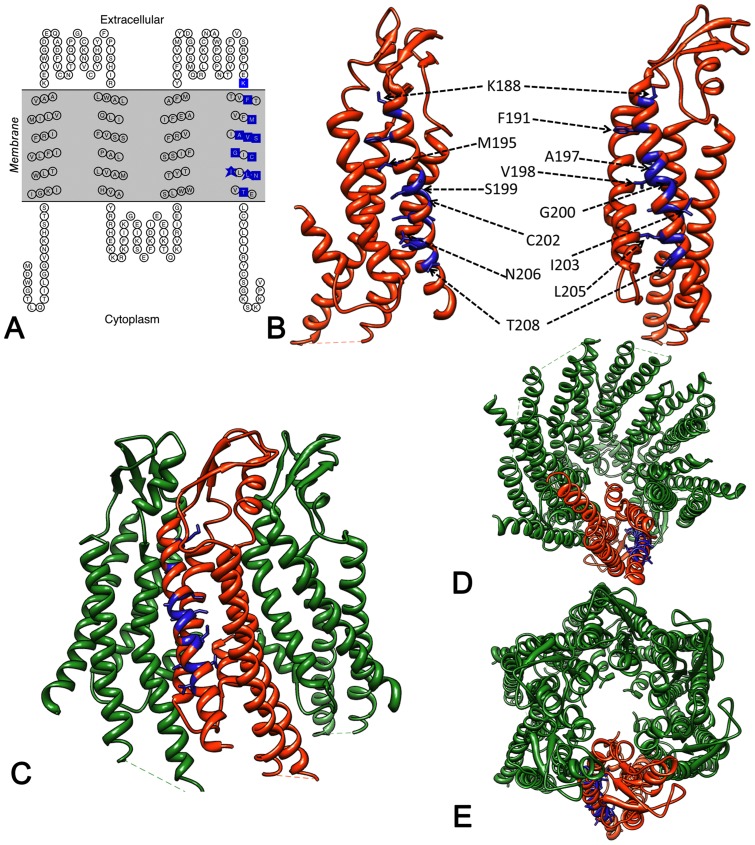
Spatial arrangement of 12 NSHL residues in TM4. (**A**) Topology diagram of Cx26. Blue boxes indicate these 12 residues. (**B–E**) Positions of residues on current X-ray atomic model (PDB ID 2ZW3) marked in blue on (**B**) monomer (**C–E**) hexamer. There is an ∼54° angle along the y axis between the two monomer views in (B). (**C**) Side view of front three connexins. Views down cytoplasmic side (**D**) and extracellular side (**E**).

The topology diagram in Fig. 1A was generated using the ExPASy Web portal (http://www.expasy.org/) to predict transmembrane domains using several algorithms and was drawn using TOPO2 [29]. We used the UCSF Chimera package [30] http://www.cgl.ucsf.edu/chimera for visualization of the spatial localization of these residues using the current Cx26 X-ray atomic model [5], PDB accession number 2ZW3 and MiFit [31] was used to analyze side chain interactions with atoms in other residues. For this analysis, probable contacts were those that would occur in less than a 4Å distance between atoms.

## Results

The majority of non-syndromic hearing loss (NSHL) cases are caused by mutations in the GJB2 gene that codes for Cx26 and studies have revealed that mutation type and frequency are largely dependent on ethnicity [32]. The 14 TM4 mutations we obtained from the literature and the Human Gene Mutation Database (http://www.hgmd.cf.ac.uk/ac/index.php) are as follows:

- **K188R** is a mutation found in a multicenter study realized to analyze the frequency and distribution of GJB2 mutations in North America. [33].
- **F191L** was described in a study conducted on 2000 Korean newborns with normal hearing and was found in two heterozygote individuals [34].
- **M195T** is a poorly documented mutation. However the M195 is considered to be one of the key residues for determining the proper folding [16]. As confirmed by the X-ray structure provided by Maeda et al., [5], together with other six residues (V43, W44, A39, A40 I74, W77 and F154), M195 is involved in the intra-protomer interaction within the two hydrophobic cores of Cx26 oligomers, stabilizing its structure [16].
- **A197S** was found linked to NSHL in a study conducted on Ghana's population. However, due to the small number of individuals carrying this mutation in heterozygosis, very little is known about it [17]. Interestingly, in mouse, rat and sea bass Cx26 sequences, but not in other higher vertebrate species, an S is normally found at this position.
- **V198M** has been shown to be cause of NSHL in two studies, one conducted in China [35] and one in south Iran [36].
- **S199F** has been described in several studies. A general analysis of GJB2 mutation frequency and distribution in North America showed the presence of this mutation as autosomal recessive [33]. A second study, conducted among deaf individuals in Colombia, revealed that S199F was the most frequent mutation present in patients affected by NSHL, confirming that the prevalence of a specific GJB2 mutation depends on the ethnicity [37]. The third and more recent study demonstrated that the S199F mutation localizes in the cytoplasm [38].
- **G200R** was found in a study conducted on 200 families from Iran affected by NSHL. In this study, the point mutation G200R was found only in homozygous individuals [39].
- **C202F**, a point mutation, in the TM4 domain, is due to a heterozygous substitution that affects the correct assembly of the channel and its functionality [18].
- **I203T and I203K** mutations have been observed in the GJB2 gene in several populations, where studies have been conducted to establish the cause of deafness of several individuals, both adults and newborns. These point mutations are the result of a transition 608 T→C for **I203T** and two transversions 608 TC→ AA for **I203K** in the GJB2 gene sequence, [17], [34], [40].
- **L205** is a residue conserved in all connexins across every species including human (**Table** **1**). **L205V** point mutation is the result of a 613 C→G transversion, which changes the CTG codon that codes for a Leu to a GTG codon that codes for a Val. This particular mutation was found in individuals of a Turkish population affected by NSHL. **L205P** was found in a Georgian Jewish family and was homozygous. Other amino acids are found to substitute the Leu at position 205, in other populations, and they are all, linked to NSHL [32] and [41].

**Table 1:** Comparison of sequence identities for the TM4 mutation positions among the 21 human connexin isoforms.

Cx26 amino acid	# of human isoforms for which this amino acid is identical.	Comments
K188	21	E187 also conserved in 21/21 human connexins
F191	20	Exception is L in Cx40.1
M195	17	Conserved β subgroup, except Cx25
A197	8	
V198	12	
S199	10	
G200	5	
C202	11	Conserved within β subgroup
I203	17	
L205	20	Exception is F in Cx30.2/Cx31.3
N206	15	
T208	1	Charged residue position at 209 (E = 20/21; D209 in Cx40.1)

A sequence alignment was performed for 21 human connexins and the TM4 region was examined for residue identity or conservation. The residue number in the left hand column refers to its position in Cx26, however this may not be the same in other connexins because of differences in the sizes of the CL.

- **N206S** resulted in a mild recessive deafness, where the N206S mutation was found with a deletion (35delG) [42] and [43]. N206S has previously been shown to correctly traffic to the cell membrane by Mese et al. 2008 [20]. Permeability studies showed that the transfer of anionic molecules (like Lucifer Yellow and cAMP) was not affected through the mutant channels, while permeability to cationic molecules (like ethidium bromide) was considerably reduced relatively to the WT Cx26 channels [20].
- **T208P** mutation was found in a study that assessed the hearing impairment degree with GJB2 biallelic mutations. T208P specifically was found in compound heterozygosity with W24X in two unrelated persons and only once with other GJB2 mutations [44].

As shown in Table 1, some of these amino acid positions range from being absolutely conserved (K188), strictly conserved (F191, L205), moderately conserved (M195, I203, N206), somewhat conserved (A197, S199, G200, C202, N206) to one appearing solely in human Cx26 (T208).

### 3D location of these twelve amino acids on the Cx26 atomic structure

In **Fig.** **1A** we highlight the positions of the twelve amino acids in TM4 on the current atomic model of Cx26 channels [5] (PDB accession code 2ZW3). As predicted by several topology algorithms, these residues all lay inside an area defined the membrane layer. In **Fig.** **1B**, the residue positions are highlighted in the monomer and in **Fig.** **1C–E**, in the hexamer, allowing us to understand and better visualize their interactions with either other transmembrane helices or the lipid bilayer.

For TM4, side chain interactions of these twelve amino acids are almost exclusively with either intra-connexin or with the membrane lipids (**Table** **2**). We analyzed the current X-ray crystallographic structure [5] for atom interactions within a 4Å distance. Both Ala197 and Gly200 face the lipid bilayer. Other side chains on some of these amino acid positions are partially (V198M, I203, I205) or largely (T208P) exposed to the lipid bilayer. The other eight amino acids are buried within the four helix bundle and have intra-connexin interactions with TM1, TM2 or TM3 in various combinations. The one exception is Phe191 that interacts with two residues in TM1 (Val38, Ala39) and one in TM4 (Val190) of the same monomer, but also interacts with two residues in the TM2 helix (Arg75, Leu79) of the adjacent monomer.

**Table 2:** Summary of side chain interactions and possible structural changes due to mutations.

Mutant	Side chain interactions as analyzed using current atomic model (PDB ID:2ZW3)	Possible effect of mutation
*K188R*	*Lys188 contains a completely buried side chain that forms polar interactions with the main chain and side chain oxygen atoms of Ser183 (TM3).*	*An Arg side chain requires stabilization by additional hydrogen bonds but no potential partners exist for these interactions in the space occupied by Lys. Satisfying the requirement to form additional polar interaction would lead to structural changes that might destabilize the TM4 helix.*
M195T	Met195 contains a buried side chain that is part of a hydrophobic volume that includes Trp77 (TM2), Ile35 (TM1) and Leu36 (TM1).	The reduction in side chain size for a Thr replacement would lead to the creation of a hydrophobic cavity that would be destabilizing. Interactions formed by the Thr side chain carbonyl group might be disruptive.
A197S	Ala197 Cβ atom is external and exposed to the lipid hydrocarbon chains.	The impact of replacement by the slightly larger and more polar Ser is expected to be minimal.
*F191L*	*Phe191 side chain is buried in the 4-helix bundle and makes hydrophobic interactions with Arg75 (TM2), Leu79 (TM2) from an adjacent monomer as well as amino acids Val38 (TM1), Ala39 (TM1) and Val190 (TM4) from the same monomer.*	*Substitution by the smaller Leu side chain would destabilize the structure by causing the formation of a hydrophobic cavity.*
*V198M*	*Val198 side chain is partially exposed. It interacts with the hydrophobic parts of side chains from Phe31 (TM1) and Arg32 (TM1).*	*A Met side chain is too large to fold inwards and form the same hydrophobic interactions with Phe31 and Arg32 as the Val. When rotated outward into a sterically unhindered position these interactions are lost.*
*S199F*	*Ser199 side chain is buried within a 4-helix bundle and is positioned so that it might form H-bonds with atom from Arg32 (TM1), Gln80 (TM2) and Glu147 (TM3).*	*A Phe side chain is too large to be accommodated in the space occupied by the Ser. A Phe might also disrupt existing polar interaction in this part of the structure as well as fail to make the H-bonds formed by the Ser.*
*G200R*	*Gly200 faces the lipid bilayer.*	*The lipid environment might not tolerate replacement by the charged Arg side chain.*
C202F	Cys202 side chain is buried within a hydrophobic**portion of the 4-helix bundle. The Cys side**chain interacts with the side chain of Leu28 (TM1).	A Phe side chain is much too large to be contained in the volume occupied by the Cys side chain and a substitution at this position would distort the structure. .
I203T	Ile203 side chain is partially exposed and makes**minimal contact with atoms in Ser199 and Glu147(TM3)	Replacement by a Thr side chain might possibly create an H-bond with the main chain carbonyl of Ser199 that would destabilize TM4.
*I203K*	*Ile203 side chain is partially exposed and makes minimal contact with atoms in Ser199 and Glu147(TM3)*	*Replacement of the hydrophobic Ile with the charged Lys would probably place the Lys side chain within an unfavorable hydrophobic lipid environmentTM4 might distort in order to remove the side chain from the lipid space.*
L205V	Leu205 side chain is partially exposed on surface**and makes contact with the hydrophobic side**chains Trp24 (TM1) and Leu25 (TM1).	Substitution by the smaller Val would eliminate favorable interactions with both Trp24 and Leu25.
*L205P*	*Leu205 side chain is partially exposed on surface and makes contact with the hydrophobic side chains Trp24 (TM1) and Leu25 (TM1).*	*Pro residues are helix breaker and substitution at this position would disrupt the TM4 helix. Absence of the Leu side chain would also eliminate favorable interactions with Trp25 and Leu25.*
N206S	Asn206 side chain is buried within the4-helix bundle**and makes specific polar interactions with Arg143**side chain (TM3) and the main chain oxygen of Cys202 (TM4).	A Ser side chain is not able to form the specific polar interactions that are formed by the Asn. The reduction is side chain size would also create a small cavity in the structure.
*T208P*	*Thr208 side chain is largely exposed to lipid bilayer.*	*Pro residues are helix breakers and substitution at this position would disrupt the TM4 helix.*

Based on an analysis of the current atomic model, we analyzed interactions between residues (middle column) and provide an explanation as to how these mutations affect the structure (right column). We indicate the transmembrane helix where an interacting residue is located within parentheses. Italicized mutants are mis-trafficking mutants.

### Eight Cx26 TM4 mutants failed to traffic to the plasma membrane

As the initial step in determining their dysfunction phenotype, we expressed all fourteen TM4 mutants in intercellular communication deficient mammalian cell lines. Our first key question was which of our fourteen TM4 mutants were able to traffic normally and reach the cytoplasmic membrane to form gap junctions. Mutants and WT Cx26 constructs were tagged with a GFP-4C label [45] for easy detection by fluorescence microscopy. We first expressed Cx26 mutant-GFP-4C constructs in HEK293T cells (data not shown). We then expressed similarly tagged Cx26 WT and mutants in HeLa cells, which are also communication deficient. HeLa cells showed less over-expression artifacts than HEK293T and are flatter and more adherent, qualities useful for imaging and the scrape dye loading assays described below. It should be noted that whether a mutant made gap junctions or mis-trafficked was independent of the two mammalian cell lines used for expression.

**Figure 2:**
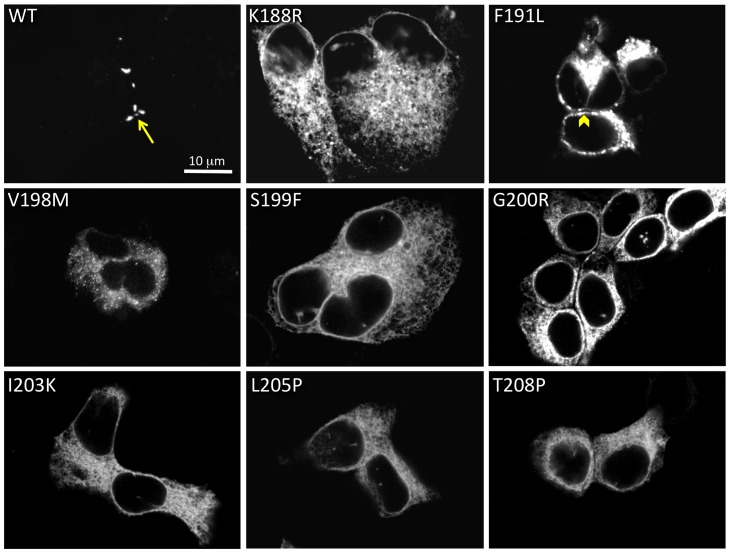
Eight TM4 deafness mutations cause mis-trafficking. All 14 mutations were tested for their ability to make gap junctions in transiently transfected HeLa cells. Eight mutations (K188R, F191L, V198M, S199F, G200R, I203K, L205P, T208P) caused mis-trafficking, typically with intracellular aggregation. WT human Cx26 is shown at the top left for comparison, with an arrow pointing to a gap junction. The arrowhead in the F191L image points to a cell-cell apposition area with aggregate fluorescence.

**Fig. 2** contains confocal images of eight mutants that showed improper trafficking. Fluorescence microscopy of WT-GFP -4C is shown for comparison, with an arrow highlighting a gap junction. The fluorescence microscopy analysis (**Fig.** **2**) confirmed previously published results that cells expressing the S199F [38] showed only cytoplasmic fluorescence. When expressed in HeLa cells, the other seven mutants (K188R, F191L, V198M, G200R, I203K, L205P and T208P) also showed intracellular fluorescence, with patterns indicating either ER retention or cytoplasmic aggregated protein. The F191L mutant formed large aggregates with some putative plasma membrane localizations, however closer inspection of these images (**Fig.** **2**) revealed that these fluorescence patterns do not have the characteristic appearance of gap junctions, since apposing plasma membranes didi not appear close enough together to make a gap junction (arrowhead).

### Six Cx26 TM4 mutants trafficked normally to the plasma membrane where they formed gap junctions

**Figure 3:**
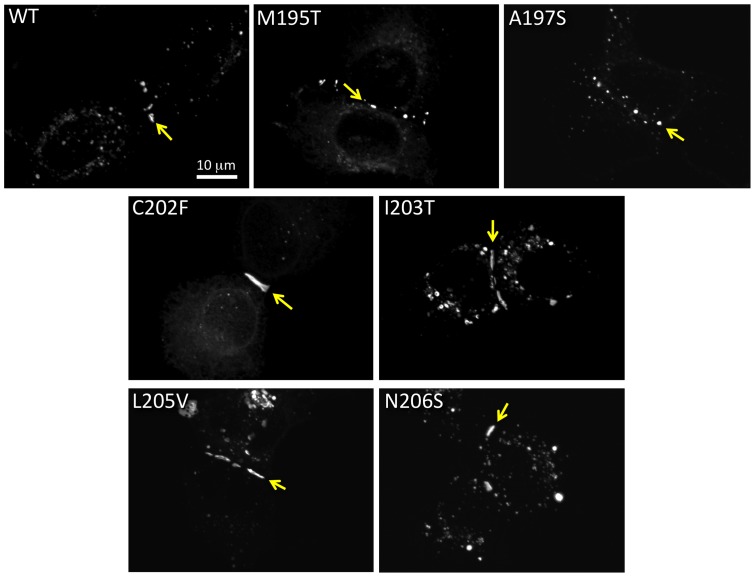
Six Cx26 TM4 traffic properly in HeLa cells. M195T, A197S, C202F, I203T, L205V and N206S each formed gap junctions between apposing cells when transiently expressed in mammalian cells. An arrow points to a gap junctions in each image.

We found that M195T, A197S, C202F, I203T, L205V and N206S mutants tagged with GFP-4C (**Fig.** **3**) did traffic normally to the plasma membrane and made gap junctions. These mutants had much less GFP fluorescence in intracellular compartments and formed gap junctions that appear as dots or short line segments between two apposing cells (**Fig.** **3** arrows). Two of these six mutations have been analyzed previously in Cx26 or Cx30. Our results are consistent with previous reports that C202F and N206S would traffic normally to the plasma membrane and localize to regions of cell apposition [16], [43], [46], [47]. Here we show that M195T, A197S, I203T and L205V also make normal appearing gap junctions when expressed in mammalian cells. All these mutants showed variability in gap junction sizes, sometimes appearing like small dots (like for the WT, M195T and A197S in **Fig.** **3**) and sometimes as long lines (as for C202F, I203T, L205V and N206S in **Fig.** **3**). However, gap junction size was highly variable and did not correlate with other characteristics such as hemichannel stability or functionality, but perhaps with transfection efficiency. Taken together these data indicate that for this subset of mutants, deafness may be due to channel malfunction and not to mis-trafficking.

### Stability of mutant oligomers after solubilization

**Figure 4:**
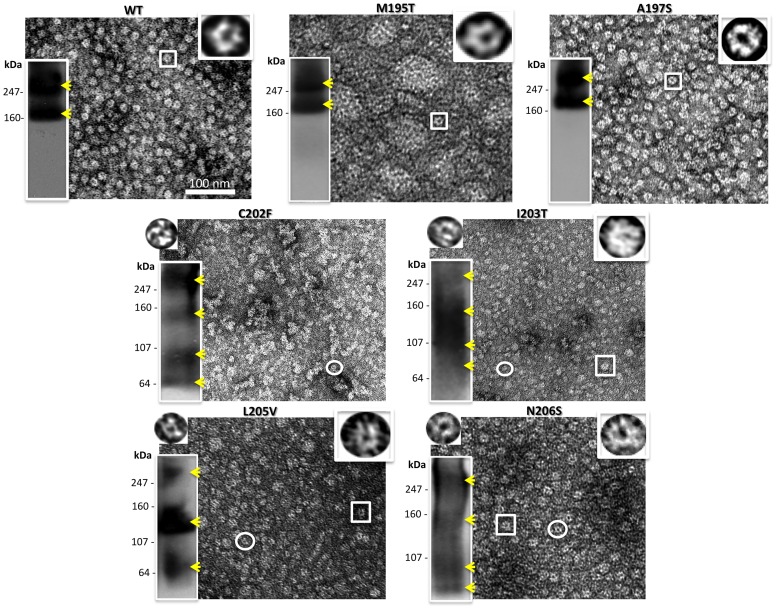
Analysis of hemichannel stability for gap junction forming mutants. Shown here are a representative electron micrograph and Blue Native westerns (bottom left hand inset) of purified hemichannels for each mutant purified from an Sf9/baculovirus expression system. The yellow arrowheads point to the recognizable bands. The insets on the top right are 6 fold enlargements of a normal hemichannel (the expected hexamer) indicated by the box in the micrograph, while the insets on the top left are 6 fold enlargements of the small oligomers visible only in unstable mutants and indicated by the circle in these micrographs.

The six gap junction forming mutants were further analyzed for their ability to make oligomers that are stable when detergent solubilized, a characteristic that we showed previously was correlated with proper functioning [21]. We tested the stability of our TM4 mutant hemichannels and channels in dodecyl maltoside, using the Sf9 insect cells-baculovirus system we described in our previous work, where we studied point mutations in the TM1, TM2 and TM3 domains of Cx26 [21]. Purified Cx26 oligomers were maintained in detergent after isolation from the lipid membranes. As in our previous study, we used EM and Blue Native gel analysis to characterize detergent stability by visual examination of preparations for “doughnut-like” structures and the presence of hexamer or dodecamer bands on native (non-denaturing) gels, respectively. Typically in our preparations, hexamers are the predominant population with a minor population of dodecamers for stable Cx26 mutants. The mutants A197S and M195T maintained stable complexes after purification, appearing indistinct from WT both in EM images and on Blue Native Western blots. On gels they appeared containing two bands corresponding to the hexamer (Cx26+tags ∼28 kDa×6 ∼ 168 kDa) and dodecamer (∼ 336 kDa), top row **Fig.** **4**).

In contrast, C202F, I203T, L205V and N206S hemichannels were less stable. Electron micrographs of these mutant preparations showed a heterogeneous population of at least two different sizes of hemichannels (**Fig.** **4**), while the Blue Native (BN) Western analysis showed extra distinct bands corresponding to dimeric, trimeric, tetrameric and pentameric macromolecular complexes. It is important to point out here, that for unstable GJ forming mutants, the bands we observed in these Blue Native Westerns varied between different preparations and gel conditions. Nonetheless, some preparations were resolved as a ladder of bands (like C202F, L205V and N206S in **Fig.** **4**) with different oligomers detectable, but others appeared as smears (like the I203T in **Fig.** **4**). Interestingly, for three of our six TM4 GJ forming mutants, EM images consistently revealed a high tendency to aggregate: the two unstable C202F, L205V oligomers and, surprisingly, connexons of the stable mutant M195T.

### Scrape Dye Loading Permeability Assays for Gap Junction forming TM4 mutants showed decreased dye transfer

**Figure 5:**
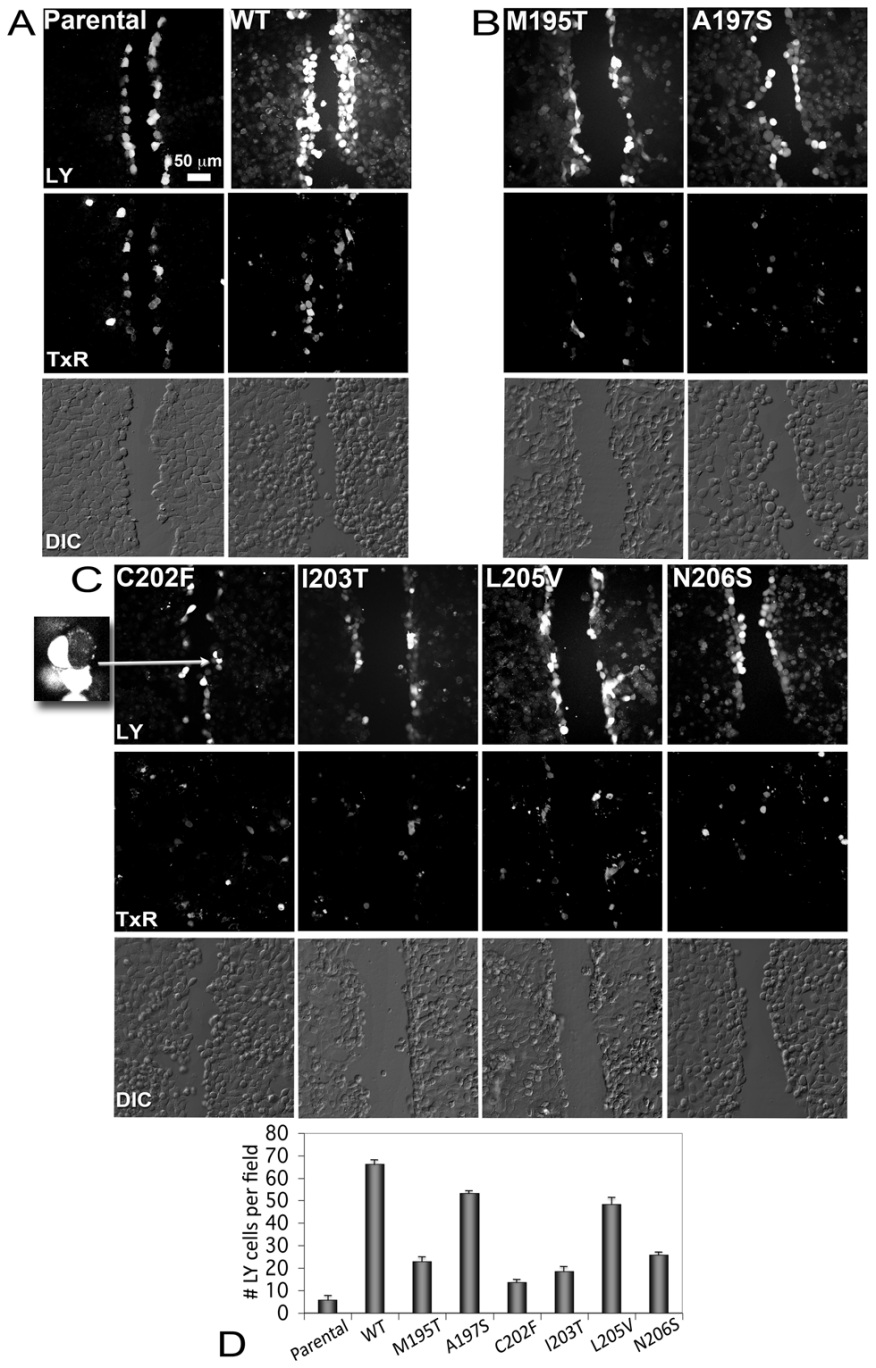
Dye transfer assays for gap junction forming mutants. Intercellular communication was assayed by scrape dye loading using Lucifer Yellow (**LY**) and Dextran Texas Red (**TxR**). LY will pass through gap junctions while Dextran TxR will not. (**Panel A**) Confluent gap-junction deficient parental HeLa cells (left column) and HeLa cells transiently transfected with Cx26 WT-GFP-4C (right column) serve as negative and positive controls, respectively. Top row: Images of LY fluorescence. Middle row: Images of TxR fluorescence Bottom row: Differential Interference Contrast (DIC) light micrograph of the same field of cells showing cell-cell contact. Dextran Texas Red acts as a reporter for dead or non-communicating cells and those along the scratch that also contained Lucifer Yellow were not counted in this analysis. (**Panel B**) Similar images as in (Panel A) for the two mutants, M195T and A197S, which made detergent stable hemichannels and channels. (**Panel C**) LY (top), TxR (middle) and DIC images (bottom) for mutants that made detergent unstable hemichannels and channels (left to right, C202F, I203T, L205V and N206S). The inset at the left is a 4.5x magnification of a LY non-transferring cell expressing C202F gap junctions. The arrow points to these cells in the C202F LY image. (**D**) Histogram of levels of communicating cells for each mutant. Error bars are standard errors of the mean (SEM).

In order to test the function of gap junctions formed by our Cx26 TM4 mutants to pass a molecule such as the Lucifer Yellow, we tested them using the scrape dye load transfer assay. This assay provides a simple, quantifiable first approximation to comparing gap junction functionality between mutants and WT. Gap junction communication deficient HeLa cells were transfected at high expression levels with WT and the six mutants that localized to intercellular junctions (M195T, A197S, C202F, I203T, L205V and N206S) as demonstrated with the GFP-4C tag. Parental HeLa cells have a low expression of dispersed connexin45 channels that allows cells to pass Lucifer Yellow in insignificantly small amounts such that they are considered communication incompetent. Thus, this parental HeLa cell line served as a negative control for comparison with transfected Cx26 mutant HeLa cells [48]. We used an established protocol for scrape loading using Lucifer Yellow dye transfer in presence of fluorescent Dextran Texas Red to label non-transferring or broken cells along the scrape [26], [27] as shown in **Fig.** **5A, 5B and 5C**. In **Fig.** **5A** top panel (labeled as LY for Lucifer Yellow), we show dye transfer between Parental HeLa cell monolayers (negative control) and transfected WT cell monolayers (positive control). While in the first case only the layer closer to the scrape contained the LY, the WT cell monolayer showed a broader transfer to distant cell layers, highlighting a very high efficiency of the WT gap junctions in passing the dye between communicating cells (as indicated in the graph in **Fig.** **5D**). The middle panel in **Fig.** **5A** as well as in **B** and **C** shows the Dextran Texas Red (TxR) channel, used to label dead cells along the scrape, that were subtracted from the LY containing cells to generate the data in the graph in **Fig.** **5D**. The bottom panel in **Fig.** **5A, B and C** are DIC (Differential Interference Contrast) images that show that cells were confluent. All the mutants differed somewhat from WT gap junctions in their ability to transfer dye (**Fig.** **5A, 5B, 5C and 5D**). As previously shown [46], we confirmed that C202F-expressing cells displayed a significantly impaired ability to pass the Lucifer Yellow (**Fig.** **5C, 5D**), with a level close to that of non-transfected cells. The mutants I203T, M195T and N206S also showed significantly decreased dye transfer (**Fig.** **5B, 5C and 5D**), at levels slightly higher than the negative control, while A197S and L205V gap junctions efficiently passed dye, albeit slightly reduced as compared to WT (**Fig.** **5B, 5C and 5D**).

### Electrophysiological Characterization of Gap Junction-Forming Mutants

**Figure 6:**
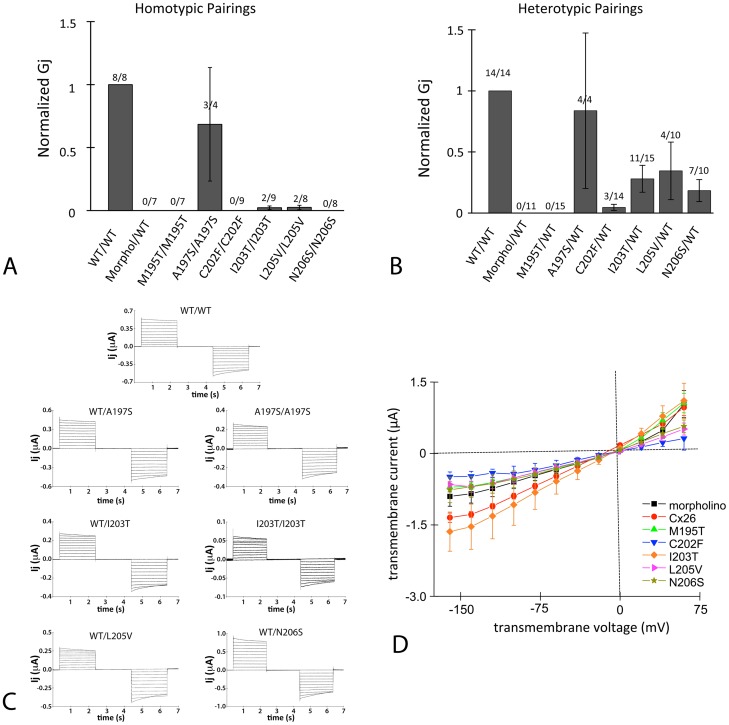
Six mutant channels (M195T, A197S, C202F, I203T, L205V and N206S) were tested for their conductances in the paired *Xenopus* oocyte system. (**A**) Mutant channels were tested in the homotypic configuration. Conductance measurements from two oocyte batches were pooled, each bar represents the mean ± SEM. The numbers above each bar represent the number of oocyte pairs in which coupling was observed as a fraction of the number tested. The negative-control (morpholino) involved oocytes injected only with the standard anti-XeCx38 antisense and intercellular conductance (Gj) was normalized to that of WT. Each bar represents a mean ± SEM and the numbers above each bar represent the number of oocyte pairs in which coupling was observed as a fraction of the number tested. (**B**) Mutants were paired heterotypically with WT and intercellular conductance (Gj) was normalized to that of WT. Conductance measurements from three oocyte batches were pooled, each bar represents the mean ± SEM, and the numbers above each bar represent the number of oocyte pairs in which coupling was observed as a fraction of the number tested. The negative-control (morpholino) involved oocytes injected only with the standard anti-XeCx38 antisense. (**C**) Characteristics of gap junctions induced by four of the six mutants after expression in Xenopus oocytes. Characteristic currents induced by WT are displayed on top Oocyte pairs were clamped at −20 mV and currents were recorded from a continuously clamped oocyte while its partner was pulsed in 10 mV increments to induce transjunctional voltages (Vj's) of up to ±100 mV. Only A197S and I203T formed distinguishable homotypic channels and these are observed on the right, adjacent to their corresponding heterotypic currents. (**D**) Transmembrane currents were measured in single oocytes to determine if mutants formed functionally conductive hemichannels. Each point represents the mean current (± SEM) for three oocytes from the same batch. The negative-control (morpholino) involved oocytes injected only with the standard anti-XeCx38 antisense.

For a more quantitative analysis of gap junction function, the paired *Xenopus* oocyte expression system was used to assess the function and properties of six mutants M195T, A197S, C202F, I203T, L205V and N206S**.** Oocytes were injected with cRNA encoding Cx26 WT or a TM4 mutant and paired overnight. Untagged Cx26 constructs were used as tags that are genetically appended tag to connexins may not affect macroscopic properties, but can affect gating, conductance properties or trafficking kinetics [49]–[51]. The following day, the dual cell two-electrode voltage clamp technique was used to assess GJIC. WTCx26 consistently induced coupling of paired oocytes, with properties similar to those reported previously for human Cx26 [1], [8], [23]. The intercellular conductance of paired oocytes expressing Cx26 WT ranged from 1 µS to 50 µS with mild sensitivity to transjunctional voltage (Fig 6C). Mutants were tested in homotypic pairings and in heterotypic pairings with WT. When paired homotypically only the A197S mutant induced significant intercellular conductance between paired oocytes (**Fig.** **6A**). Two other mutants I203Tand L205V induced GJIC occasionally, at levels much lower than Cx26 WT (**Fig** **6A**) and with voltage-sensitivity closely resembling Cx26 WT (see I203T/I203T, **Fig.** **6C**). Applying a student's t-test to the I203T/I203T and L205V/L205V pairings, each is significantly different from Cx26 WT with a p<0.05.

**Fig. 6B** summarizes intercellular conductance for heterotypically paired mutants. While A197S induced robust coupling in oocytes, M195T appeared completely non-functional. The three other mutants (I203T, L205V, N206S) formed heterotypic channels fairly consistently, but with conductance levels at least 50% lower than WT homotypics. In light of data showing that all mutants localize to intercellular junctions (Fig 2), the electrophysiological data suggests that M195T is unable to form conductive channels despite its localization to junctions, that A197S functions similarly to WT and that the other mutants (C202F, I203T, L205Vand N206S) may create populations of hemichannels with normal gating properties but reduced availability for GJIC. Evidence for this comes from the observation that conductance levels are lower than those of WT in heterotypic pairings and further reduced in homotypic pairs. In all cases, mutants gated similarly to WTCx26, whether paired homo- or heterotypically (**Fig.** **6C**). Hence, the six mutations tested in the oocyte expression system do not appear to alter the voltage-gating mechanisms of gap junction channels.

Because the ability of mutants to form hemichannels with physiologically significant conductance levels has been correlated with disease [52], the ability of several Cx26 M4 deafness mutants to form hemichannels was tested using single oocytes injected with the same amount of RNA, and incubated for the same duration, as oocytes used for coupling experiments. **Fig.** **6D** shows the average transmembrane current (µA) versus transmembrane voltage (Vm) for a set of experiments involving M195T, C202F, I203T, L205V and N206S. Current levels across a wide range of voltages were similar to those of the negative control oocytes (injected with morpholino antisense oligonucleotide against XeCx38) and were small compared to those induced by gap junction proteins that formed functional hemichannels. For instance, the Cx26 hemichannel-forming mutant G45E induced outward currents of about 8000 nA at +60 mV in oocytes, a level over 8-fold higher than WT in the same study [53]. This suggests that hemichannel formation was not responsible for disease phenotypes associated with these mutations.

### Detergent stability of heteromers of M195T, C202F, I203T, L205V or N206S with WT

In our previous work [21], co-infecting insect cells with a baculovirus that contained the DNA for hCx26-mutant with an appended V_5_-His_6_ tag, and with a baculovirus containing WT (no tag), allowed us to isolate heteromeric oligomers formed by both mutated and WT connexins. The theory behind this experiment is that, based on the ratios of the multiplicity of infection (MOI) of the virus used for infection, the two connexin species (mutant and WT, with and without tag respectively) would be expressed by the cells and since the mutant connexin by itself was incapable of forming only homomeric hexamers, or dodecamers, those oligomers, detected after co-purification, would be heteromeric (WT no tag and mutant-V5-His_6_ tag). We previously found that purifying WT-no tag, gave us no connexin monomers or oligomers detectable either by EM or BN Westerns. The co-purification of mutated and WT protein allowed us to observe that while the WT was able to rescue the hemichannel stability for certain point mutations, it failed or even further destabilized the oligomers for others [21]. Here we applied this “rescuing tool” to all our unstable mutants and to the M195T, in order to investigate if our unstable mutants could become stable and at what WT/mutant ratio and if the aggregation tendency of the M195T mutant would disappear.

**Figure 7:**
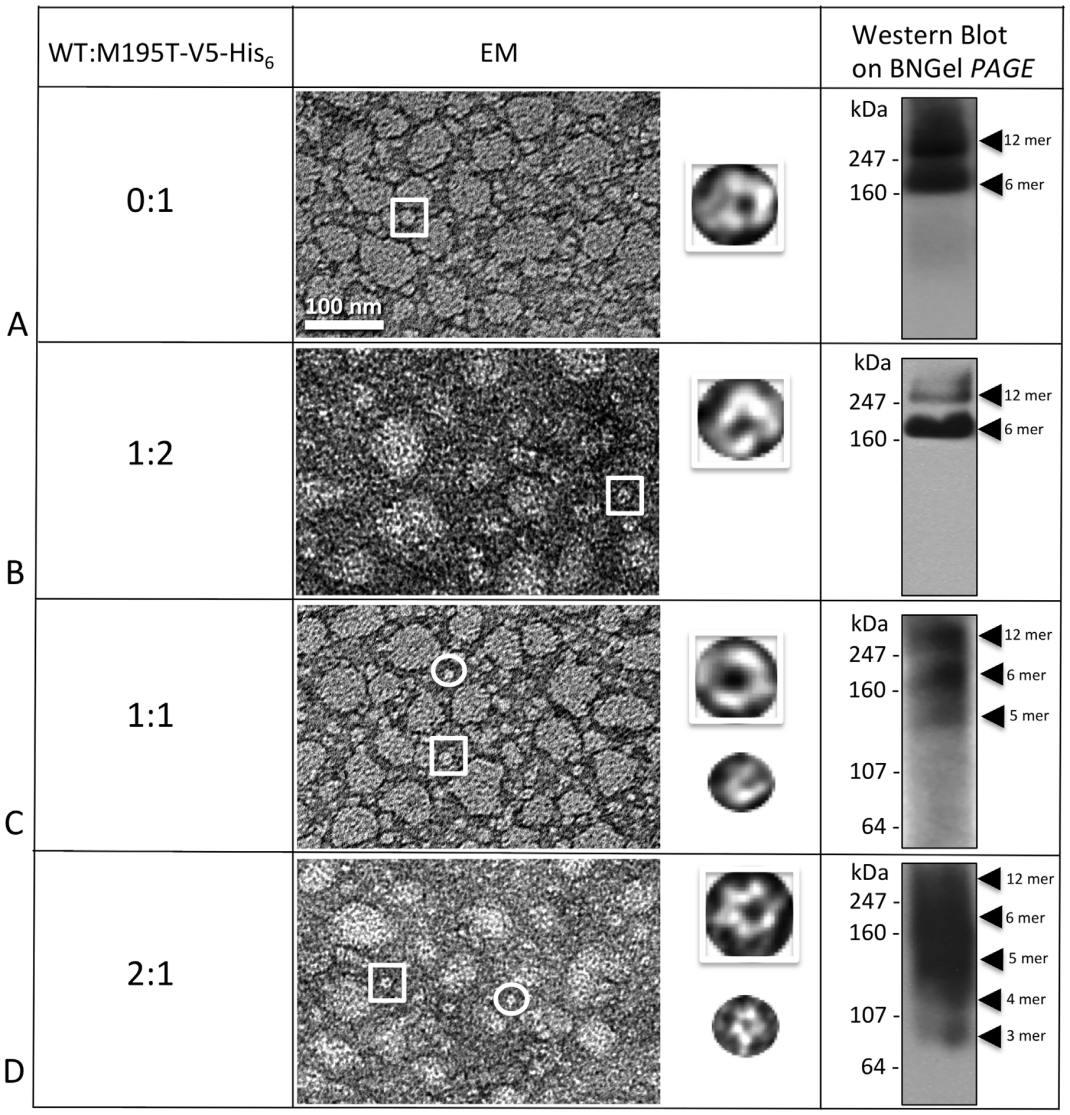
Co-expression of WT with M195T–WT decreases the stability of heteromers. (**A–D**) Hemichannels formed by M195T and the WT analyzed in different ratios showed increased instability from heteromeric interactions. EM images showed consistent aggregation of hemichannels for all four ratios analyzed, from 0:1 to 2:1. The Blue Native (BNGel PAGE) westerns confirmed stability with only two bands (hexamer and dodecamer) for the ratios 0 and 1:2 (*A* and *B*), but higher ratios of WT:M195T-V5-His_6_ are unstable with (**C**) three predominant bands for 1:1 and (**D**) a ladder of bands for the 2:1 ratio.

In **Fig. 7**, the analyses performed on M195T hemichannels demonstrated that M195T hemichannels and channels are stable by biochemical analysis, however EM images revealed significant aggregation. In these micrographs, image fields contained large clumps of hemichannels, mixed with smaller aggregates and single hemichannels (**Fig.** **7A**). For the 1:2 M195T/WT ratio (**Fig.** **7B**), where there is a smaller starting population of the M195T monomers, while heteromeric hemichannels/channels remained aggregated, as visible by EM, the BN Western contained only two bands, one corresponding to the dodecamer and the other to the hexamer. The BN Western of WT/M195T 1:1 ratio preparations showed an extra band around the size of a WT pentamer plus a light smear below this band (**Fig.** **7C**). The WT/M195T 2:1 ratio (**Fig.** **7D**) confirmed even further this instability effect of the WT monomers on the heteromeric M195T/WT hemichannels. In these WT/M195T 2:1 preparations, the aggregation was far more severe in EM images and the BN Western detected faster migrating connexin species. We concluded that the addition of WT subunits to M195T hemichannels, not only did not improve the aggregation tendency of this mutant, but even increased instability at the ratio 1:1 and even more so at 2:1.

**Figure 8:**
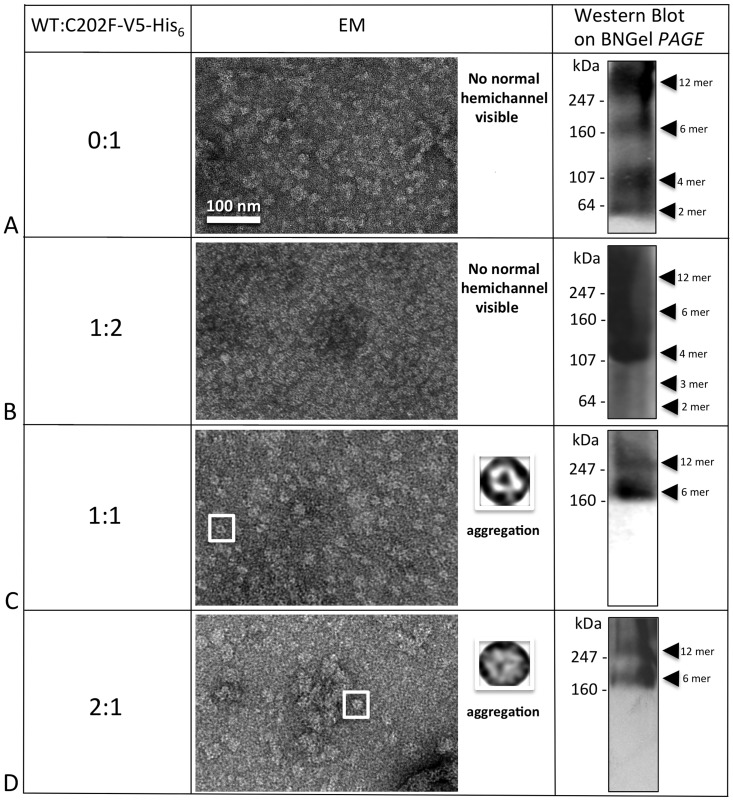
C202F hemichannel stability can be rescued by the WT. (**A**) C202F forms unstable oligomers not easily recognizable by EM as hemichannels and a ladder of bands as detected by Blue Native western analysis. (**B**) The addition of WT protomers to the mutant oligomers did not result in stable hemichannels for the 0:1 ratio neither by EM or Blue Native western analysis. (**C,** **D**) The higher ratios (1:1 and 2:1) clearly showed only two bands on Blue Native westerns at the hexamer and dodecamer positions and more recognizable doughnut-like structures in EM images, but these heteromeric channels tended to aggregate.

We also found that the unstable C202F mutant (**Fig.** **8A**) could be successfully rescued. While the 1:2 WT/C202F ratio failed to restore hemichannel/channel structures (**Fig.** **8B**), both the WT/C202F ratios of 1:1 (**Fig.** **8C**) and 2:1 (**Fig.** **8D**) successfully rescued channel structure and oligomer states of hexamers and dodecamers. BN Westerns consistently exhibited two bands for the 1:1 and 2:1 ratios (**Fig.** **8C and 8D**) and EM images revealed channel structures more similar to WT. For the 2:1 ratio, aggregation was the most severe for this mutant, confirming the importance of the TM4 domain in the interaction with other monomers for the structure stability.

**Figure 9:**
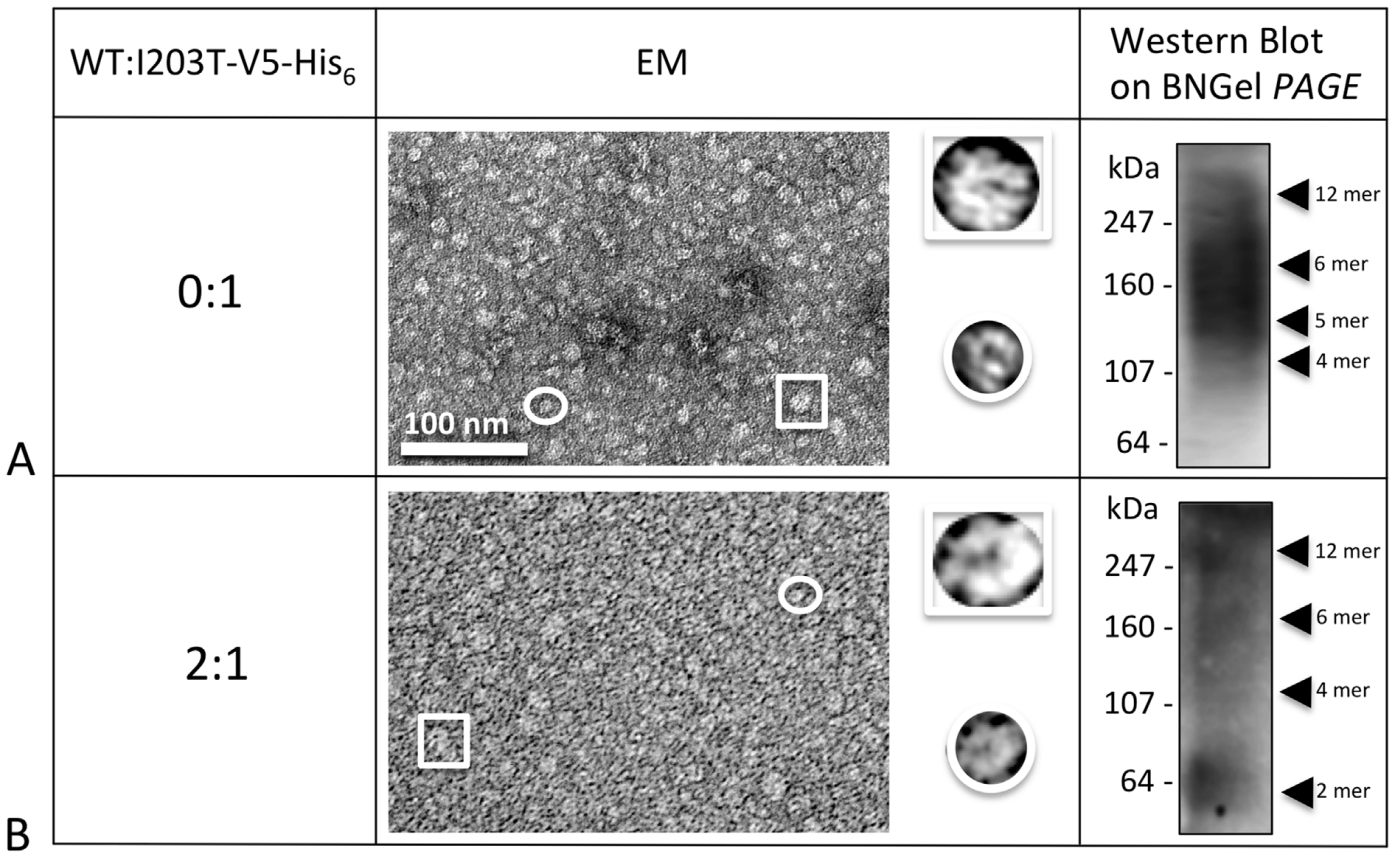
I203T mutant stability cannot be rescued by the WT. (**A**) Homomeric I203T hexamers and dodecamers are unstable. Both EM and Blue Native western analyses clearly showed no rescuing the I203T mutant hemichannels, even at the very high ratio of 2 (**B**), where the majority of the heteromer subunits should contain WT.

**Figure 10:**
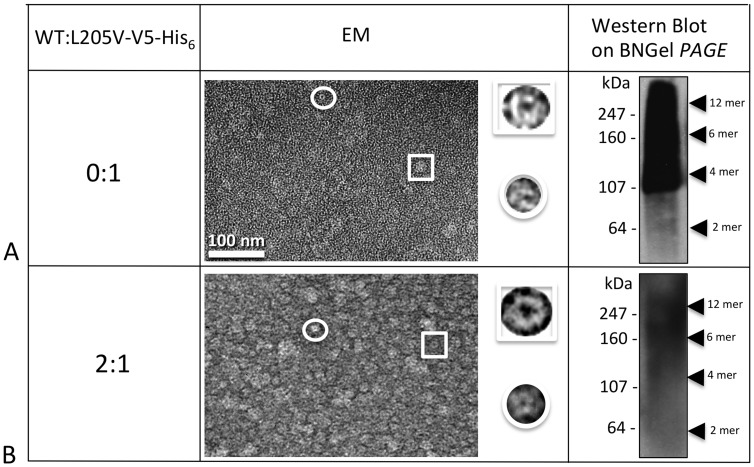
L205V mutant cannot be rescued by the WT. (**A**) Homomeric oligomers of L205V are unstable. Similar to the I203T mutant shown in Fig. 9, (**B**) this mutant did not display stable hemichannels or channels when I203T monomers where mixed with the WT monomers in a 2:1 ratio of WT:L205V-V5-His_6_.

Hemichannel/channel structures for two mutants, I203T and L205V, could not be rescued by the WT (**Figs.** **9** **,** **10**). For I203T, the co-expression of WT in a ratio 2:1 caused oligomers to become even more unstable as indicated by the EM and the BN Western results. For L205V, co-expression with WT did not rescue proper oligomerization (**Fig.** **10B**).

**Figure 11:**
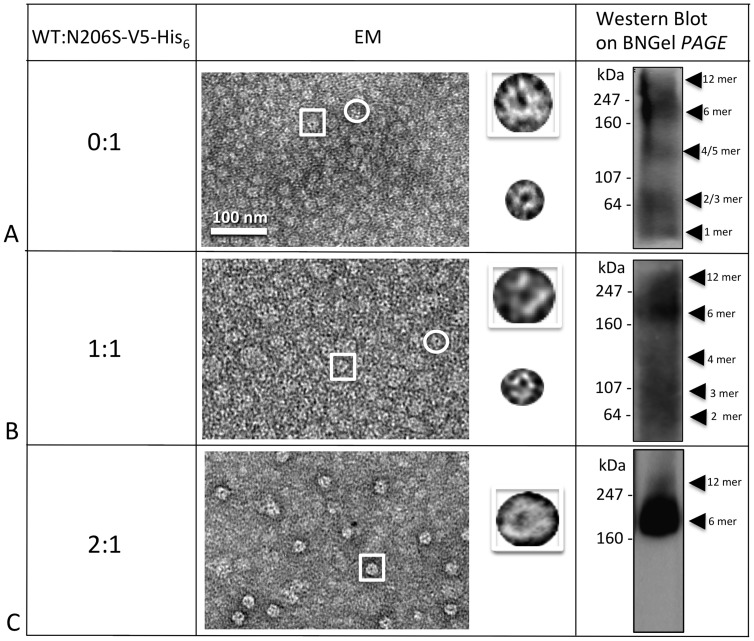
N206S forms stable hemichannels when expressed with WT. **(A–C)** As visible by both EM and Blue Native Western analysis, stable hemichannels only resulted at 2:1 ratios of WT:N206S-V5-His_6_ (**C**). The EM image clearly showed distinct hemichannel structures and the hexamer band predominates in the Blue Native (BNGel PAGE) Western (C).

Finally, in **Fig.** **11** we report the successful rescuing of stable N206S mutant hemichannels by the WT. The mutant N206S can be rescued by the WT only in the WT/N206S 2:1 ratio, suggesting that WT protomers are necessary in higher ratios as compared to mutant in order to form the heteromeric hemichannel. The 1:1 ratio of WT/N206S showed non-homogenous channels in EM images and a ladder of bands for the BN Western, while the ratio 2:1 reconstituted nicely shaped channels that were confirmed to be either hexamers or dodecamers by BN Western blot (**Fig.** **11**). The hexamer population predominates within these preparations.

## Discussion

It is well established that connexin defects or mutations are linked to several diseases referred to as connexinopathies. These connexin mutations cause pathogenesis in a tissue specific manner and the mechanism for this is still largely unclear [54]. Gap junction mediated intercellular communication is essential for the auditory function [16] and Cx26 is the gap junction protein most associated with the NSHL and SHL forms of deafness. In our study here, we tried to establish if there is any correlation between hemichannel (connexon) instability and defective functionality of Cx26 in point mutations, in TM4, that are found to be responsible for cases of NSHL [16]–[18], [20], [32], [34], [40]–[43]. The goals of our study would be to understand how mutations in TM4 affect structure, stability and interactions with other helix and lipid interfaces, whether this is correlated to function and to establish if the mutation would be compensated in the structure by WT, as could happen with heterozygous patients. In **Table** **3** we have summarized our results, describing which Cx26 mutants resulted stable (M195T, A197S) and unstable (C202F, I203T, L205V and N206S). In **Table** **3** are also listed the mutants that can be rescued by the WT (C202F and N206S) and ones that cannot (I203T and L205V) as well as the eight trafficking mutants. Taken together these results demonstrate the critical consequences of substitutions at these 12 residues found in deaf patients.

**Table 3:** Summary of non-trafficking mutant characteristics from this study.

Mutant	GJs in**HeLa	Stable hemi-channels	Stable heteromeric**hemichannels	Dye Passage	Current Passage	
					Homo-typic	Hetero-typic
M195T	Yes	Yes	Causes aggregation	Significantly reduced	No	No
A197S	Yes	Yes	NT	Little less than WT	Little less than WT	Little less than WT
C202F	Yes	No	Yes	Significantly reduced	No	No
I203T	Yes	No	No	Significantly reduced	No	Significantly reduced
L205V	Yes	No	No	Yes, but below WT levels	Significantly reduced	Significantly reduced
N206S	Yes	No*	Yes	Significantly reduced	Significantly reduced	No

NT  =  not tested, because homomers are stable. *  =  weakly unstable.

### Cx26 TM4 domain is important for both protein folding and functionality

As shown in this study and in previously published ones [10], [15], the fourth transmembrane domain of Cx26 is very important for the hemichannel/channel stability and functionality that any mutation in this region will cause different folding and conformational changes. More recent discoveries such as the X-ray structure published by Maeda and colleagues clearly demonstrate that residues in this domain have critical inter-connexin associations with two of the three other helices. Key interactions between protomers to form a hemichannel are mostly located in the extracellular half of transmembrane helices TM4 and TM2 as well as in extracellular loops. Specifically, some residues that sit in the TM4 area such as M195 would play an important role in the intra-monomer interactions, but also stabilize the entire channel structure [16], an essential feature for proper functioning.

### TM4 mutants display various phenotypes in trafficking, stability and function

**Figure 12:**
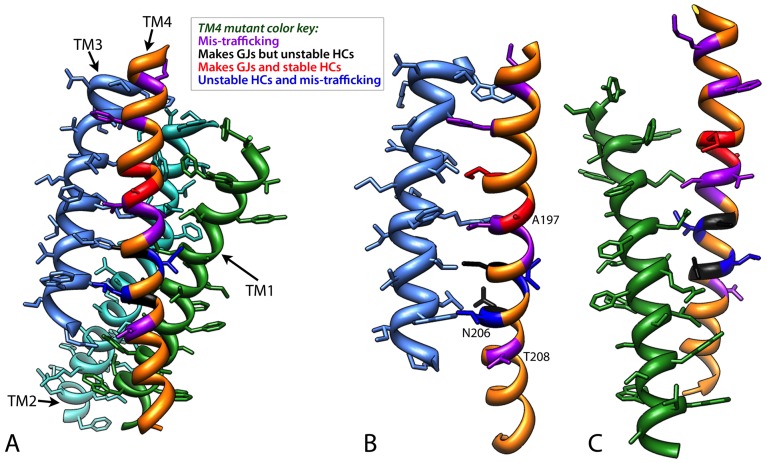
Mapping of the twelve TM4 residues on the current X-ray model for the four transmembrane helices. Since the twelve amino acid positions analyzed fall only in the four α helical bundle domain of Cx26, here we show only (**A**) TM1 (medium blue), TM2 (cyan) TM3 (green) and TM4 (orange). The orientation of the Cx26 subunit is the same as in Fig. 1 with the extracellular loops at the top of the image. The mutant residues have been colored coded such that mutations that caused -trafficking are shown in purple, mutations that make gap junctions but unstable hemichannels are black and mutations that make stable gap junctions and hemichannels are indicated in red. Two positions, I203 and L205, have different phenotypes for the each of two mutations at these positions. These residues are dark blue. (**B**) Side chain interactions for these residues are between TM4 and TM1 and (**C**) TM4 and TM3. Note that the A197, N206S and T208 face the lipid bilayer. Note the mutant phenotypes are not mapped to any particular face of TM4.

While it is sometimes more straightforward in understanding how gap junction forming mutations in side chains facing the pore such as ones in TM1–TM3 or within the extracellular loops could affect function and hemichannel/channel formation, it is less understood how TM4 mutations influence function, particularly those facing the lipid bilayer (**Table** **2** **,** **Fig.** **12** **, Movie S1**). The phenotypes of these mutations are not easily predicted by the current static atomic structure and therefore, experimental testing is still necessary to determine at what stage (synthesis, trafficking, docking and functionality), human Cx26 mutants are defective in intercellular communication.

Since many of these mutants had only been identified by a genetic analysis of the patient, we tested each mutant for proper trafficking in mammalian cells. After transiently transfecting gap junction deficient HeLa cells with our mutated Cx26 tagged by GFP, we were able to detect gap junctions for six out of fourteen mutants. It should be noted that two amino acids, I203 and L205, had two mutations at their position. For both these amino acid positions where the side chains are partially exposed to the lipids, the two mutations caused two different phenotypes with one being mis-trafficked (I203K and L205P), while I203T and L205V form non-functional hexamers and dodecamers. For these two sets of mutants it is clear that a radical change in side chain, such as a replacement with a charge or helix breaking ability, caused intracellular aggregation, most likely due to aberrant folding. For presumably I203T and L205V, these mutations are compensated by shifts in helix positions but function is eliminated or diminished.

For mutants that formed gap junctions, scrape dye loading assays revealed that each mutant had different dye passing capabilities. A197S and L205V hemichannels were able to pass the Lucifer Yellow, even if in a smaller amount than the WT, while the rest of the mutants had little or no dye transfer. In the oocyte expression system, M195T channels failed to induce intercellular coupling, while C202F channels had a severely impaired ability to form channels. Failure of M195T to make functional channels in Xenopus oocytes versus in mammalian cells may be due to the change in hydrophilicity of this channel resulting in different interactions with the lipids of Xenopus oocyte plasma and internal membranes as opposed to mammalian cells. It is worth noting that Xenopus oocytes are typically maintained at lower temperatures than mammalian cells, a variable that would affect membrane fluidity as well. Alternatively, the M195T mutant channel may have distinct permeability properties that are different from their conductance properties. In contrast, A197S forms channels robustly and with features very similar to those of WT Cx26. The other mutants (I203T, L205V and N206S) are capable of forming channels with properties similar to those of WT, but do so with reduced efficiency, possibly due to a reduction in the availability of hemichannels capable of forming intercellular channels.

In order to determine which of the six Cx26 mutants formed stable structures, we purified these in a baculovirus/Sf9 insect cell expression system. After dissolving the membrane fractions where hemichannels/channels are embedded, only two mutants, M195T and A197S remained stable after detergent solubilization, showing well defined hemichannel structures similar to WT. The BN Western blots confirmed these two mutants stability detecting only two bands corresponding to the hexamer and dodecamer. All the other mutant connexins/channels (C202F, I203T, L205V and N206S) were unstable as determined by EM and BN Western blots. However, it is important to note that there are degrees of instability whereby N206S oligomers appear more stable with distinct smaller oligomers in BN Westerns versus I203T channels/hemichannels that were the most unstable, as evidenced by the smear of staining in the BN Westerns. This oligomer instability in detergent is indicative of mutant channels that can form when held together by lipid bilayers either in gap junctions or single membranes but have weaker intra-monomer interactions than WT. Most of the time, this weakness is correlated with a decrease in functionalities (dye transfer or currents).

Of these six gap junction forming mutants, C202F and N206S hemichannel structures could be rescued by their co-expression with the WT, while I203T or L205V mutants could not. However, heteromeric N206S/WT hemichannels/channels revealed donut-shaped structures, instead, inspection of heteromeric C202F/WT preparations by EM showed aggregates that made recognition of WT-like structures difficult. A197S hemichannels/channels were stable and very similar to the WT. This result is not unexpected given that a Ser is normally found at position 197 in rodent species.

Interestingly, M195T mutant hemichannels had a high tendency to aggregate like no other stable mutant. We speculate that this tendency to aggregate can be explained by the fact the M195T mutation increases hydrophilicity, since in the presence of detergent, hemichannels tend to “escape” from it, by forming large clumps. Adding double distilled water to freshly made hemichannel preparations, always restored a less aggregated appearance with orientations perpendicular to channel axis at the EM, looking very similar to WT hemichannels. We also found very interesting that the M195T aggregation tendency could not be eliminated by the WT and instead the resulting WT/M195T heteromeric channels were unstable. Adding to our observations that M195T was impaired in functional assays for intercellular communication such as passing of current or dye, these observations are consistent with the interpretation that M195T is part of a hydrophobic cavity and the prediction that a “hole” would occur from a Thr and lead to protein rearrangements (**Table** **2**).

## Conclusions

Our studies described here confirmed the importance of the TM4 region of Cx26 in the hemichannel structure and in channel functionality. Except for the A197S, all our mutants showed either structure instability or reduced functionality or both. In this work, we were able to show that NSHL mutations changed interactions with other helices and the lipid bilayer that correlated with decreased functionality. Understanding how mutant proteins influenced some of these interactions could possibly explain why deafness occurs in either homozygous or heterozygous cases and its severity. In theory, this tool may be beneficial in determining whether a gene therapy approach could be successfully applied to deaf patients with specific Cx26 mutations to mitigate their effects. A gene therapy approach whereby preparations of an antisense oligonucleotide to correct defective USH1C protein was successfully applied to cochleas in knockout mice generated to have symptoms similar to Usher syndrome, another hereditary child deafness syndrome [55].

## Supporting Information

Movie S1 This movie shows an animation of the four helix bundle (TM1-TM4) of a connexin monomer with the colors for the different mutant phenotypes as in Fig. 12. A TM2 from an adjacent monomer is also shown for interactions with Phe191.
(MOV)

## References

[1] Sohl G, Willecke K (2004) Gap junctions and the connexin protein family. Cardiovasc Res 62: 228–232. 15094343 10.1016/j.cardiores.2003.11.013

[2] Harris A, Locke D (2009) Connexins : a guide. New York, N.Y.: Springer. xvi, 573 p.

[3] Saez JC, Berthoud VM, Branes MC, Martinez AD, Beyer EC (2003) Plasma membrane channels formed by connexins: their regulation and functions. Physiol Rev 83: 1359–1400. 14506308 10.1152/physrev.00007.2003

[4] Evans WH, Martin PE (2002) Gap junctions: structure and function (Review). Mol Membr Biol 19: 121–136. 12126230 10.1080/09687680210139839

[5] Maeda S, Nakagawa S, Suga M, Yamashita E, Oshima A, et al (2009) Structure of the connexin 26 gap junction channel at 3.5 A resolution. Nature 458: 597–602. 19340074 10.1038/nature07869

[6] Oshima A, Tani K, Hiroaki Y, Fujiyoshi Y, Sosinsky GE (2008) Projection structure of a N-terminal deletion mutant of connexin 26 channel with decreased central pore density. Cell Commun Adhes 15: 85–93. 18649181 10.1080/15419060802013588 PMC2527467

[7] Oshima A, Tani K, Hiroaki Y, Fujiyoshi Y, Sosinsky GE (2007) Three-dimensional structure of a human connexin26 gap junction channel reveals a plug in the vestibule. Proc Natl Acad Sci U S A 104: 10034–10039. 17551008 10.1073/pnas.0703704104 PMC1886001

[8] Oshima A, Tani K, Toloue MM, Hiroaki Y, Smock A, et al (2011) Asymmetric configurations and N-terminal rearrangements in connexin26 gap junction channels. J Mol Biol 405: 724–735. 21094651 10.1016/j.jmb.2010.10.032 PMC3026138

[9] Pfenniger A, Wohlwend A, Kwak BR (2011) Mutations in connexin genes and disease. Eur J Clin Invest 41: 103–116. 20840374 10.1111/j.1365-2362.2010.02378.x

[10] Xu J, Nicholson BJ (2013) The role of connexins in ear and skin physiology – Functional insights from disease-associated mutations. Biochim Biophys Acta 1828: 167–178. 22796187 10.1016/j.bbamem.2012.06.024 PMC3521577

[11] Cohen JMM, Gorlin RJ (1995) Epidemiology, etiology, and genetic patterns. In: Gorlin RJ, Toriello HV, Cohen Jr MM, editors. Hereditary Hearing Loss and Its Syn dromes. New York: Oxford Monographs on Medical Genetics. 9–21.

[12] Dror AA, Avraham KB (2010) Hearing impairment: a panoply of genes and functions. Neuron 68: 293–308. 20955936 10.1016/j.neuron.2010.10.011

[13] Unger VM, Kumar NM, Gilula NB, Yeager M (1999) Three-dimensional structure of a recombinant gap junction membrane channel. Science 283: 1176–1180. 10024245 10.1126/science.283.5405.1176

[14] Sosinsky GE, Nicholson BJ (2005) Structural organization of gap junction channels. Biochim Biophys Acta 1711: 99–125. 15925321 10.1016/j.bbamem.2005.04.001

[15] Lee JR, White TW (2009) Connexin-26 mutations in deafness and skin disease. Expert Rev Mol Med 11: e35. 19939300 10.1017/S1462399409001276

[16] Wang WH, Liu YF, Su CC, Su MC, Li SY, et al (2011) A novel missense mutation in the connexin30 causes nonsyndromic hearing loss. PLoS One 6: e21473. 21731760 10.1371/journal.pone.0021473 PMC3123352

[17] Hamelmann C, Amedofu GK, Albrecht K, Muntau B, Gelhaus A, et al (2001) Pattern of connexin 26 (GJB2) mutations causing sensorineural hearing impairment in Ghana. Hum Mutat 18: 84–85. 10.1002/humu.1156 11439000

[18] Morle L, Bozon M, Alloisio N, Latour P, Vandenberghe A, et al (2000) A novel C202F mutation in the connexin26 gene (GJB2) associated with autosomal dominant isolated hearing loss. J Med Genet 37: 368–370. 10807696 10.1136/jmg.37.5.368 PMC1734593

[19] Zainal SA, Md Daud MK, Abd Rahman N, Zainuddin Z, Alwi Z (2012) Mutation detection in GJB2 gene among Malays with non-syndromic hearing loss. Int J Pediatr Otorhinolaryngol 76: 1175–1179. 22613756 10.1016/j.ijporl.2012.04.027

[20] Mese G, Valiunas V, Brink PR, White TW (2008) Connexin26 deafness associated mutations show altered permeability to large cationic molecules. Am J Physiol Cell Physiol 295: C966–974. 18684989 10.1152/ajpcell.00008.2008 PMC2575827

[21] Ambrosi C, Boassa D, Pranskevich J, Smock A, Oshima A, et al (2010) Analysis of four connexin26 mutant gap junctions and hemichannels reveals variations in hexamer stability. Biophys J 98: 1809–1819. 20441744 10.1016/j.bpj.2010.01.019 PMC2862186

[22] Oshima A, Doi T, Mitsuoka K, Maeda S, Fujiyoshi Y (2003) Roles of Met-34, Cys-64, and Arg-75 in the assembly of human connexin 26. Implication for key amino acid residues for channel formation and function. J Biol Chem 278: 1807–1816. 12384501 10.1074/jbc.M207713200

[23] Elfgang C, Eckert R, Lichtenberg-Frate H, Butterweck A, Traub O, et al (1995) Specific permeability and selective formation of gap junction channels in connexin-transfected HeLa cells. J Cell Biol 129: 805–817. 7537274 10.1083/jcb.129.3.805 PMC2120441

[24] Martin BR, Giepmans BN, Adams SR, Tsien RY (2005) Mammalian cell-based optimization of the biarsenical-binding tetracysteine motif for improved fluorescence and affinity. Nat Biotechnol 23: 1308–1314. 16155565 10.1038/nbt1136

[25] Gaietta G, Deerinck TJ, Adams SR, Bouwer J, Tour O, et al (2002) Multicolor and electron microscopic imaging of connexin trafficking. Science 296: 503–507. 11964472 10.1126/science.1068793

[26] Govindarajan R, Zhao S, Song XH, Guo RJ, Wheelock M, et al (2002) Impaired trafficking of connexins in androgen-independent human prostate cancer cell lines and its mitigation by alpha-catenin. J Biol Chem 277: 50087–50097. 12205082 10.1074/jbc.M202652200

[27] Boassa D, Solan JL, Papas A, Thornton P, Lampe PD, et al (2010) Trafficking and recycling of the connexin43 gap junction protein during mitosis. Traffic 11: 1471–1486. 20716111 10.1111/j.1600-0854.2010.01109.x PMC3272544

[28] Skerrett IM, Merritt M, Zhou L, Zhu H, Cao F, et al (2001) Applying the Xenopus oocyte expression system to the analysis of gap junction proteins. Methods Mol Biol 154: 225–249. 11218651 10.1385/1-59259-043-8:225

[29] Johns, SJ (nd) TOPO2, Transmembrane protein display software web site. Available: http://www.sacs.ucsf.edu/TOPO2/. Accessed 2012 Dec 12.

[30] Pettersen EF, Goddard TD, Huang CC, Couch GS, Greenblatt DM, et al (2004) UCSF Chimera--a visualization system for exploratory research and analysis. J Comput Chem 25: 1605–1612. 15264254 10.1002/jcc.20084

[31] McRee DE (2004) Differential evolution for protein crystallographic optimizations. Acta Crystallogr D Biol Crystallogr 60: 2276–2279. 15572781 10.1107/S0907444904025491

[32] Yilmaz A, Menevse S, Bayazit Y, Karamert R, Ergin V, et al (2010) Two novel missense mutations in the connexin 26 gene in Turkish patients with nonsyndromic hearing loss. Biochem Genet 48: 248–256. 19941053 10.1007/s10528-009-9314-7

[33] Putcha GV, Bejjani BA, Bleoo S, Booker JK, Carey JC, et al (2007) A multicenter study of the frequency and distribution of GJB2 and GJB6 mutations in a large North American cohort. Genet Med 9: 413–426. 17666888 10.1097/gim.0b013e3180a03276

[34] Han SH, Park HJ, Kang EJ, Ryu JS, Lee A, et al (2008) Carrier frequency of GJB2 (connexin-26) mutations causing inherited deafness in the Korean population. J Hum Genet 53: 1022–1028. 19043807 10.1007/s10038-008-0342-7

[35] Yuan Y, You Y, Huang D, Cui J, Wang Y, et al (2009) Comprehensive molecular etiology analysis of nonsyndromic hearing impairment from typical areas in China. J Transl Med 7: 79. 19744334 10.1186/1479-5876-7-79 PMC2754984

[36] Hashemi SB, Ashraf MJ, Saboori M, Azarpira N, Darai M (2012) Prevalence of GJB2 (CX26) gene mutations in south Iranian patients with autosomal recessive nonsyndromic sensorineural hearing loss. Mol Biol Rep 39: 10481–10487. 23073770 10.1007/s11033-012-1929-9

[37] Tamayo ML, Olarte M, Gelvez N, Gomez M, Frias JL, et al (2009) Molecular studies in the GJB2 gene (Cx26) among a deaf population from Bogota, Colombia: results of a screening program. Int J Pediatr Otorhinolaryngol 73: 97–101. 19027181 10.1016/j.ijporl.2008.10.001

[38] Xiao Z, Yang Z, Liu X, Xie D (2011) Impaired membrane targeting and aberrant cellular localization of human Cx26 mutants associated with inherited recessive hearing loss. Acta Otolaryngol 131: 59–66. 20863150 10.3109/00016489.2010.506885

[39] Chaleshtori MH, Rad HL, Dolati M, Sasanfar R, Hoseinipour A, et al (2005) Frequencies of mutations in the connexin 26 gene (GJB2) in two populations in Iran (Tehran and Tabriz). Iranian J Publ Health 34: 1–7.

[40] Kudo T, Ikeda K, Kure S, Matsubara Y, Oshima T, et al (2000) Novel mutations in the connexin 26 gene (GJB2) responsible for childhood deafness in the Japanese population. Am J Med Genet 90: 141–145. 10607953 10.1002/(sici)1096-8628(20000117)90:2<141::aid-ajmg10>3.0.co;2-g

[41] Leshinsky-Silver E, Berman Z, Vinkler C, Yannov-Sharav M, Lev D (2005) A novel missense mutation in the Connexin 26 gene associated with autosomal recessive sensorineural deafness. Hear Res 202: 258–261. 15811717 10.1016/j.heares.2004.11.003

[42] Wu BL, Lindeman N, Lip V, Adams A, Amato RS, et al (2002) Effectiveness of sequencing connexin 26 (GJB2) in cases of familial or sporadic childhood deafness referred for molecular diagnostic testing. Genet Med 4: 279–288. 12172394 10.1097/00125817-200207000-00006

[43] Kenna MA, Wu BL, Cotanche DA, Korf BR, Rehm HL (2001) Connexin 26 studies in patients with sensorineural hearing loss. Arch Otolaryngol Head Neck Surg 127: 1037–1042. 11556849 10.1001/archotol.127.9.1037

[44] Snoeckx RL, Huygen PL, Feldmann D, Marlin S, Denoyelle F, et al (2005) GJB2 mutations and degree of hearing loss: a multicenter study. Am J Hum Genet 77: 945–957. 16380907 10.1086/497996 PMC1285178

[45] Gaietta GM, Giepmans BN, Deerinck TJ, Smith WB, Ngan L, et al (2006) Golgi twins in late mitosis revealed by genetically encoded tags for live cell imaging and correlated electron microscopy. Proc Natl Acad Sci U S A 103: 17777–17782. 17101980 10.1073/pnas.0608509103 PMC1635977

[46] Yum SW, Zhang J, Scherer SS (2010) Dominant connexin26 mutants associated with human hearing loss have trans-dominant effects on connexin30. Neurobiol Dis 38: 226–236. 20096356 10.1016/j.nbd.2010.01.010 PMC2868926

[47] Zhang J, Scherer SS, Yum SW (2011) Dominant Cx26 mutants associated with hearing loss have dominant-negative effects on wild type Cx26. Mol Cell Neurosci 47: 71–78. 21040787 10.1016/j.mcn.2010.10.002 PMC3132585

[48] Hülser DF, Rehkopf B, Traub O (1997) Dispersed and aggregated gap junction channels identified by immunogold labeling of freeze-fractured membranes. Experimental Cell Research 233: 240–251. 9194487 10.1006/excr.1997.3568

[49] Bukauskas FF, Bukauskiene A, Bennett MV, Verselis VK (2001) Gating properties of gap junction channels assembled from connexin43 and connexin43 fused with green fluorescent protein. Biophys J 81: 137–152. 11423402 10.1016/S0006-3495(01)75687-1 PMC1301499

[50] Desplantez T, Halliday D, Dupont E, Severs NJ, Weingart R (2011) Influence of v5/6-His tag on the properties of gap junction channels composed of connexin43, connexin40 or connexin45. J Membr Biol 240: 139–150. 21424225 10.1007/s00232-011-9352-z PMC3069306

[51] Jordan K, Solan JL, Dominguez M, Sia M, Hand A, et al (1999) Trafficking, assembly, and function of a connexin43-green fluorescent protein chimera in live mammalian cells. Mol Biol Cell 10: 2033–2050. 10359613 10.1091/mbc.10.6.2033 PMC25409

[52] Lee JR, Derosa AM, White TW (2009) Connexin mutations causing skin disease and deafness increase hemichannel activity and cell death when expressed in Xenopus oocytes. J Invest Dermatol 129: 870–878. 18987669 10.1038/jid.2008.335 PMC6463483

[53] Gerido DA, White TW (2004) Connexin disorders of the ear, skin, and lens. Biochim Biophys Acta 1662: 159–170. 15033586 10.1016/j.bbamem.2003.10.017

[54] Scott CA, Kelsell DP (2011) Key functions for gap junctions in skin and hearing. Biochem J 438: 245–254. 21834795 10.1042/BJ20110278

[55] Lentz JJ, Jodelka FM, Hinrich AJ, McCaffrey KE, Farris HE, et al.. (2013) Rescue of hearing and vestibular function by antisense oligonucleotides in a mouse model of human deafness. Nat Med. 10.1038/nm.3106 PMC3657744 23380860

